# Us vehicles sales. Evidence of persistence after COVID-19

**DOI:** 10.1371/journal.pone.0281906

**Published:** 2023-02-21

**Authors:** Gema Lopez, Luis Alberiko Gil-Alana

**Affiliations:** 1 Universidad Francisco de Vitoria, Madrid, Spain; 2 University of Navarra, Pamplona, Spain; Newcastle University, UNITED KINGDOM

## Abstract

In this paper, the sales of vehicles in the US are examined to understand if the shock caused by the current COVID-19 pandemic has had permanent or transitory effects on its subsequent evolution. Using monthly data from January 1976 until April 2021 and fractional integration methods, our results indicate that the series reverts and the shocks tend to disappear in the long run, even when they appear to be long lived. The results also indicate that the COVID-19 pandemic has not increased the degree of persistence of the series but, unexpectedly, has slightly reduced its dependence. Thus, shocks are transitory, long lived but, as time goes by, the recovery seems to be faster, which is possibly a sign of the strength of the industry.

## 1. Introduction

The analysis of US vehicles sales has multiple benefits and can help with understanding a range of aspects, such as the behaviour of the buyers of the product, the relationship between sales and external elements that influence the sector’s companies (for example: logistical issues, raw material shortages, fluctuation in fuel prices, etc.) and the existence of possible crises that could be of a general nature or be industry or company specific. An industry view is wider and deeper than an analysis purely of companies or brands. It gives us information about the US vehicles market that we can use to create hypotheses about future°market movements that can affect, for example, share prices and are relevant to investors.

In terms of data, sales are key in industry performance [1]. Sales data shows the success of the products offered, the existence of customer demand and that there is sufficient purchasing power to buy such products or services. Data has showed us that the development of the industry usually increases sales over time, and the brands that do not manage to evolve with the market or the demands of society disappear. This is what happened with many analogue products that were affected by new technology, such as: cameras, video tapes or telephones. This behaviour corresponds to what we know as the product life cycle [2].

**Fig 1:**
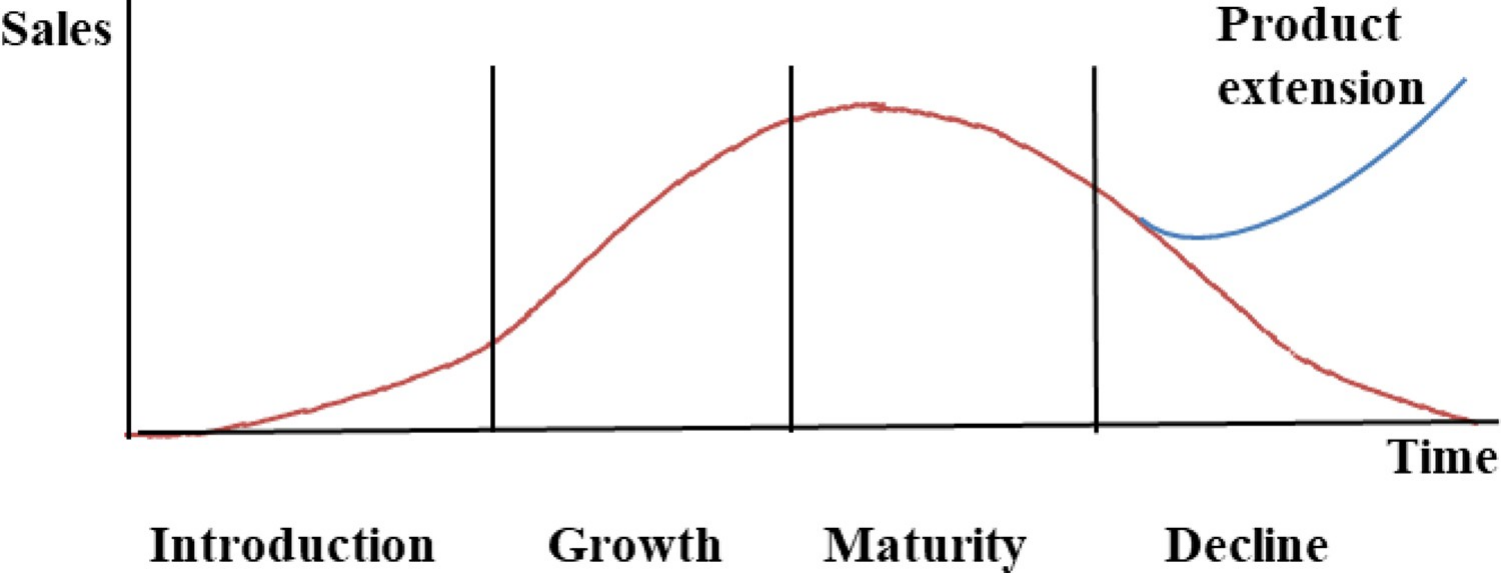
Product life cycle graphic.

As Fig 1 shows, we can distinguish 4 clear phases. All the phases’ names are very descriptive:

- Introduction: when the product or service is introduced into the market. The investment per unit or service sold is high while the sales numbers are low. There is a significant effort on promotion and sales strategies.
- Growth: the product or service is already known in the market and accepted and sales increase over time. The investment per unit or service sold is more moderate (although typically higher in absolute terms) and the income is rising with lower proportional effort than in the previous phase.
- Maturity: at this stage there are other competitors, and the product or service is well established in the market. Growth is difficult and the only way to increase income is to start thinking of new markets and or new product extensions. This moment is key in the company. It is necessary to evolve with the market because otherwise the Decline phase will be entered, which ultimately would lead to the disappearance of the product.
- Decline: the last one. All products/services reach this point as they are impacted by any combination of new technology, evolving needs, new competitors, etc. As highlighted in the previous phase, the key is to anticipate trends in the market and develop accordingly to offset the decline in the original business.

The launch of new product variations (in packaging, product formula, uses, etc.) enables the company’s ongoing presence in the market, as well as covers new buyer needs or preferences developing in the market, thus enhancing the competitiveness of the company in mature markets where differentiation and innovation are key to success [3].

Thanks to this ongoing process, an industry such as the automobile industry has been able to flourish, and we can analyse more than 40 years of sales data. This gives sufficient data to understand new cycles and possible behaviour.

There are clear reasons for choosing the car industry and the US market specifically. The evolution of this sector’s importance to the US economy has always been strong, historically contributing 3–3.5 percent to the overall Gross Domestic Product (GDP) [4]. In 2020, the US automotive industry contributed 3% to the US Gross Domestic Product [5].

There is no doubt that the general performance of the automotive sector has been strong, above all over the last few years. It increased employment and disposable income, which, together with a favourable stock market, have helped foster a generally positive perception of the strength of the US economy [6].

Our interest in analysing this sector came from this significant contribution and the desire to understand how the pandemic might influence its evolution. While, on the one hand, it would be logical to think that private transport won the battle against public transport during the pandemic because people were afraid of contact and crowded places [7, 8], on the other hand, the decline in the purchasing power of some groups would not allow the acquisition of these goods, which take up a significant amount of the annual home budget and are often not a priority [9]. In the end, what counts are the numbers and, in our case, the sales data. They give us clarity on the reality of the market and, in this case, are positive. According to Boudette [10], car sales helped get the American economy off to a good start in 2021. There could be many reasons for the improvement in 2021: Biden’s strategy for expanded support for electric vehicles [11], the development of the electric cars category [12] or that people could prefer travelling by car versus planes given the latter’s lower reliability at that time with so many cancelations, restrictions and uncertainty [13]. In this article, we will look even further into this shock, trying to understand if that increase has a temporary or a permanent effect on the series.

The significance of the market was also the reason that we chose to analyse the US. The US market leads norms, laws, sanctions and market rules that are followed by other markets or countries. Its requirements and management make it an undeniable reference in the global industry. Its economic and industrial leadership gives it this role [14].

Having explained the data used and our reasons for choosing the industry and geography, we can introduce our methodology, fractional integration. This analysis will help to understand the past to figure out possible future behaviours of the market, which has also been used in other articles such as Quineche [15]. In Section 3, data and methodology, we explain the system used and its benefits.

Anticipating briefly our results, we obtain that the series reverts and the observed shocks tend to disappear in the long run, even when they appear long term. The results show that the COVID-19 pandemic has not increased the degree of persistence of the series but, unexpectedly, has slightly reduced its dependence. Thus, shocks are transitory, long lived but, as time goes by, the recovery seems to be faster, which is possibly a sign of the strength of the industry.

The paper is structured as follows: Section 2 includes a short review of the literature. Section 3 describes the dataset and the methodology used in the paper. Section 4 focuses on the empirical results, while Section 5 contains some conclusions.

## 2. Literature review

Having introduced the paper and the database, it is necessary to understand previous works by other authors. As we have described, the automobile sector is important and extremely big, so we can find much literature on the topic, ranging from the types of vehicles, consumer approach, trade, supply chain and manufacturing to the consequences of the use of its products. Some examples are as follows.

Modelling studies have been used to understand the consumer preferences for alternative fuel vehicles [16]. The structural time series model has been employed for data over the period 1949–2019 allowing for both asymmetric price responses and an underlying energy demand trend for gasoline [17]. The effects of vehicle safety design on road traffic deaths, injuries, and the public health burden have been analysed by a modelling study by Bhalla and Gleason [18], applied to the Latin American region. Hamed and Al-Eideh [19] investigate the demand for electric vehicles using two approaches, one related to Information Indices and the second based on Poisson regression models, showing the importance of modelling this issue. As one can see, the industry is vast and there are many aspects to analyse.

While we can find an immense number of articles on the automobile sector, analyses on the topic of sales in that industry are much more limited. To find a general review we have to go back to 2004 [20], when a study was carried out on the regional nature of the world’s automotive sector, and to 2012 for a vector error correction model to analyse automobile sales forecasting [21]. We go back to 2011 for a study in the US and the Japanese markets, analysing forecasts of car sales in the US and forecasts of car registrations in Japan [22]. For China, we find a few more articles. Huo and Wang [23] tried modelling future vehicle sales and stock in China by 2050. More recently, in 2017 we find another one about forecasting electric vehicles sales [24] and in 2018 an econometric model was used to forecast Chinese automobile sales [25]. Also in 2017, and changing market to Greece, we find a real-time vector autoregressive analysis by Konstantakis et al. [26] for modelling the dynamic response of automobile sales in troubled times. In the case of Russia, Solntsev et al. [27] analyse sales in light weight vehicles and the results obtained enabled improvements in the forecast of the promotion / introduction of selected brands of passenger vehicles in the Russian market in a reasonable way with the aim of subsequently justifying the possibility of developing an Inventory Management System for spare parts and materials. With this review we conclude that the topic of our article is bringing new analysis and information to the industry related to its sales in the US, together with an explanation about its historical and possible future behaviour.

Regarding the methodology, most of the above-mentioned papers focus on traditional time series methods that only consider integer degrees of differentiation. That is, first differences are adopted if the series is nonstationary, in which case the series is said to be integrated of order 1, or I(1), or no differences are adopted if the series is stationary I(0). In this paper we depart from these strong assumptions by allowing fractional degrees of differentiation. Fractional integration methods belong to the category of long memory models and they have been previously used in the analysis and modelling of sales, such as in the neural network methodology employed by Bandara et al. [28] to forecast sales demand in e-commerce, in the long short-term memory (lstm) based sales forecasting model of Goel and Bajpai [29] and in a seasonal long memory model to analyse time series forecasting of agricultural products’ sales volumes [30]. It is important not to lose sight of the main objective of any organisation, to survive. That is why hitting the sales target is its main task and the reason why companies focus their resources and efforts to predict sales, take decisions to increase them or at least remain stable [31]. In addition, fractional integration permits us to determine if exogenous shocks in the series have transitory or permanent effects depending on the value of the differencing parameter. Thus, if this number is smaller than 1, shocks will have a transitory nature and the series will return to their original trend faster as lower the value is; on the other hand, if the order of integration is equal to or higher than 1, the effect of the shock will be permanent, requiring actions on the series to return to their original long-term projection.

## 3. Data and methodology

We use monthly data on US vehicles sales from January 1976 to April 2021 (a year after the pandemic) obtained from FRED [32], short for Federal Reserve Economic Data. FRED is part of The Federal Reserve Bank of St. Louis, Economic Research Division. It is an online database consisting of hundreds of thousands of economic data time series from scores of national, international, public, and private sources.

**Fig 2:**
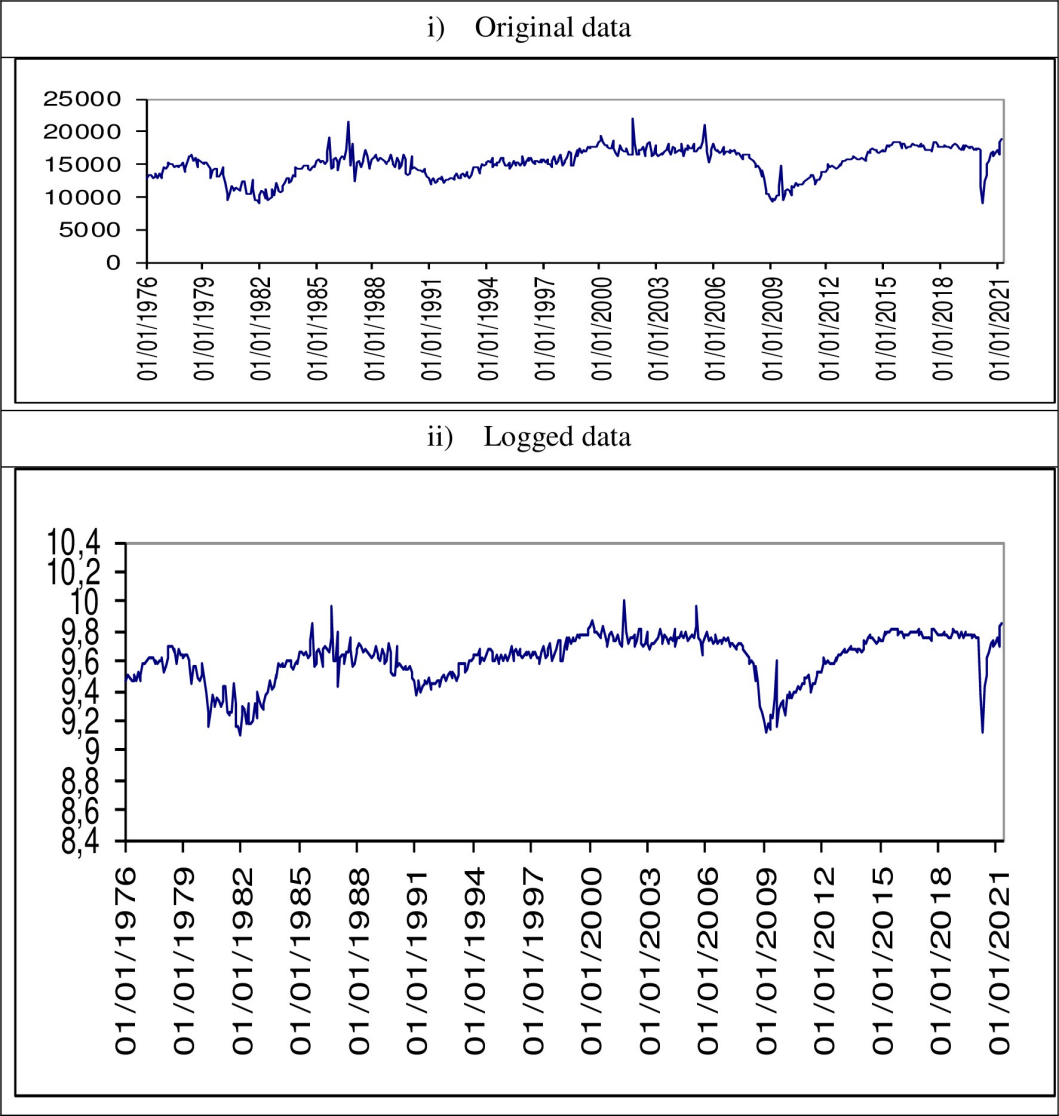
Time series data.

The time series data (in Fig 2) shows ups and downs typical of a market and in general a linear and positive evolution during these 45 years.

For the methodology, we use fractional integration, which is based on the idea that the number of differences required in a series to render it stationary I(0) may be a fractional value. This is of course more flexible that the classical approaches that simply assume integer degrees of integration (usually 1), and therefore, allows for a much richer degree of modelization of the series. This approach is also useful if we want to determine the nature of exogenous shocks in the series, which have transitory effects if the order of integration is smaller than 1, but permanent if that value is equal to or higher than 1. We now explain this point further. A process {x_t_, t = 1, 2, …] is said to be integrated of order d, and denoted by I(d) if it can be expressed as

$${\left(1-B\right)}^{d}x_{t}=u_{t},\;t=1,2,\ldots ,$$ (1)

where B is the backshift operator, i.e., Bx_t_ = x_t-1_, and u_t_ is integrated of order 0 or I(0), which may include potentially weak autocorrelation of the AutoRegressive Moving Average (ARMA)-form. Fractional integration takes place when d is a fractional number, and if u_t_ is ARMA(p,q), we say that x_t_ is an AutoRegressive Fractionally Integrated Moving Average (ARFIMA) model. In this context, a high degree of flexibility is permitted since we can include a large battery of model specifications, including i) the I(0) or short memory case, if d = 0; stationary long memory (if 0 < d < 0.5); nonstationary though mean reverting processes (0.5 ≤ d 1); unit roots (d = 1) or even explosive processes (d > 1). Also, the process in (1) can be expressed in terms of an infinite MA process, such that if d < 1, the coefficients decay to zero at a hyperbolic rate implying a long memory mean reverting pattern.

In the empirical section, the model under examination includes deterministic terms like a constant and a linear time trend, i.e.,

$$y_{t}=\beta_{0}+\beta_{1}t+x_{t};\;{\left(1-B\right)}^{d}x_{t}=u_{t,}\;t=1,2,\ldots ,$$ (2)

where y_t_ refers to the observed data, β_0_ and β_1_ are unknown coefficients referring respectively to the constant and the linear time trend, x_t_ is I(d), where d can be any real value, and u_t_ is integrated of order 0 or I(0) and will adopt various specifications to permit both uncorrelation and weak autocorrelation in the error term. The estimation is conducted via the Whittle function in the frequency domain as documented in Robinson [33]. employing alternative parametric, Sowell [34] and semiparametric GPH and Robinson [35, 36] produced almost identical results to those reported in the paper.

## 4. Empirical results

Table 1 displays the estimates of the differencing parameter, d, in Eq (1) under three potential scenarios: 1) with no deterministic terms, i.e., imposing β_0_ = β_1_ = 0 in (1); 2) with only an intercept, i.e., imposing β_1_ = 0 and estimating β_0_ and d, and 3) estimating both an intercept and a linear time trend, i.e., estimating β_0_ and β_1_ along with d in (1). The results of the upper panel refer to the original data, while those in the lower panel refer to the logged values. For each case, the values reported in the first row are based on the assumption that u_t_ in (1) is a white noise process; the second row display the results under the assumption of weak autocorrelation, in particular, imposing the non-parametric method of Bloomfield [37]; finally, and based on the monthly nature of the data, a seasonal (monthly) AR(1) process is assumed on u_t_.

**Table 1:** Estimates of the differencing parameter: 1976m1 – 2021m4.

i) Original data			
Type of Errors	No regressors	An intercept	An intercept with a liner time trend
White Noise	0.82	**0.66**	0.66
	(0.77, 0.88)	**(0.61, 0.71)**	(0.61, 0.72)
Bloomfield	0.86	**0.68**	0.68
	(0.78, 0.97)	**(0.60, 0.77)**	(0.60, 0.78)
Monthly AR(1)	0.81	**0.64**	0.65
	(0.76, 0.95)	**(0.60, 0.71)**	(0.60, 0.71)
ii) Logged data			
Type of Errors	No regressors	An intercept	An intercept with a liner time trend
White Noise	1.00	**0.69**	0.69
	(0.94, 1.06)	**(0.64, 0.75)**	(0.64, 0.75)
Bloomfield	0.99	**0.70**	0.70
	(0.90, 1.11)	**(0.62, 0.79)**	(0.62, 0.79)
Monthly AR(1)	1.00	**0.68**	0.68
	(0.94, 1.07)	**(0.63, 0.74)**	(0.63, 0.74)

In bold the selected specification for each type of disturbances. In bold, the 95% confidence bands for the estimated values of d.

We first note in Table 1 that the time trend coefficient is insignificant in the three models (white noise, Bloomfield and monthly AR(1) disturbances), and the estimated values of d are in the range [0.5., 1] in the three cases, implying nonstationary mean reverting behaviour. Similar evidence is obtained with the log-transformed data, and though the estimated values of d are now slightly higher, they are still in the range [0.5, 1] supporting nonstationarity (d ≥ 0.5) and mean reversion (d < 1). Table 2 displays the estimated coefficients on the selected models.

**Table 2:** Estimated coefficients for the selected models in Table 1: 1976m1 – 2021m4.

i) Original data			
Type of Errors	No regressors	An intercept	An intercept with a liner time trend
White Noise	**0.66**	13333.989 (16.98)	---
	**(0.61, 0.71)**		
Bloomfield	**0.68**	13270.388 (16.43)	---
	**(0.60, 0.77)**		
Monthly AR(1)	**0.64**	13368.226 (17.28)	---
	**(0.60, 0.71)**		
ii) Logged data			
Type of Errors	No regressors	An intercept	An intercept with a liner time trend
White Noise	**0.69**	9.488 (169.11)	---
	**(0.64, 0.75)**		
Bloomfield	**0.70**	9.468 (167.07)	---
	**(0.62, 0.79)**		
Monthly AR(1)	**0.68**	9.490 (171.30)	---
	**(0.63, 0.74)**		

In bold the selected specification for each type of disturbances. In bold, the 95% confidence bands for the estimated values of d.

We next repeat the experiment but this time with data ending at February 2020, that is, the last month prior to the big decrease caused by the COVID-19 pandemic. The results are reported across Tables 3 and 4. We observe that once more the time trend coefficient is found to be statistically insignificant, and the values of d are again in the [0.5, 1] interval in all cases for both the original and the log-transformed data. According to these results, the series is mean reverting and though shocks will have a long life they tend to disappear in the long run.

**Table 3:** Estimates of the differencing parameter: 1976m1 – 2020m2.

i) Original data			
Type of Errors	No regressors	An intercept	An intercept with a liner time trend
White Noise	0.78	**0.64**	0.64
	(0.73, 0.83)	**(0.60, 0.69)**	(0.59, 0.69)
Bloomfield	0.84	**0.73**	0.73
	(0.77, 0.94)	**(0.65, 0.80)**	(0.65, 0.80)
Monthly AR(1)	0.76	**0.61**	0.61
	(0.71, 0.82)	**(0.57, 0.67)**	(0.57, 0.67)
ii) Logged data			
Type of Errors	No regressors	An intercept	An intercept with a liner time trend
White Noise	0.99	**0.67**	0.67
	(0.93, 1.06)	**(0.63, 0.73)**	(0.63, 0.73)
Bloomfield	0.99	**0.75**	0.75
	(0.92, 1.09)	**(0.68, 0.83)**	(0.68, 0.83)
Monthly AR(1)	0.99	**0.65**	0.65
	(0.93, 1.07)	**(0.61, 0.71)**	(0.61, 0.71)

In bold the selected specification for each type of disturbances. In bold, the 95% confidence bands for the estimated values of d.

**Table 4:** Estimated coefficients for the selected models in Table 1: 1976m1 – 2020m2.

i) Original data			
Type of Errors	No regressors	An intercept	An intercept with a liner time trend
White Noise	0.64	134442.835 (19.08)	---
	(0.60, 0.69)		
Bloomfield	0.73	13136.863 (16.41)	---
	(0.65, 0.80)		
Monthly AR(1)	0.61	13144.518 (18.52)	---
	(0.57, 0.67)		
ii) Logged data			
Type of Errors	No regressors	An intercept	An intercept with a liner time trend
White Noise	0.67	9.492 (187.39)	---
	(0.63, 0.73)		
Bloomfield	0.75	9.478 (172.24)	---
	(0.68, 0.83)		
Monthly AR(1)	0.65	9.496 (193.03)	---
	(0.61, 0.71)		

In bold the selected specification for each type of disturbances. In bold, the 95% confidence bands for the estimated values of d.

**Fig 3:**
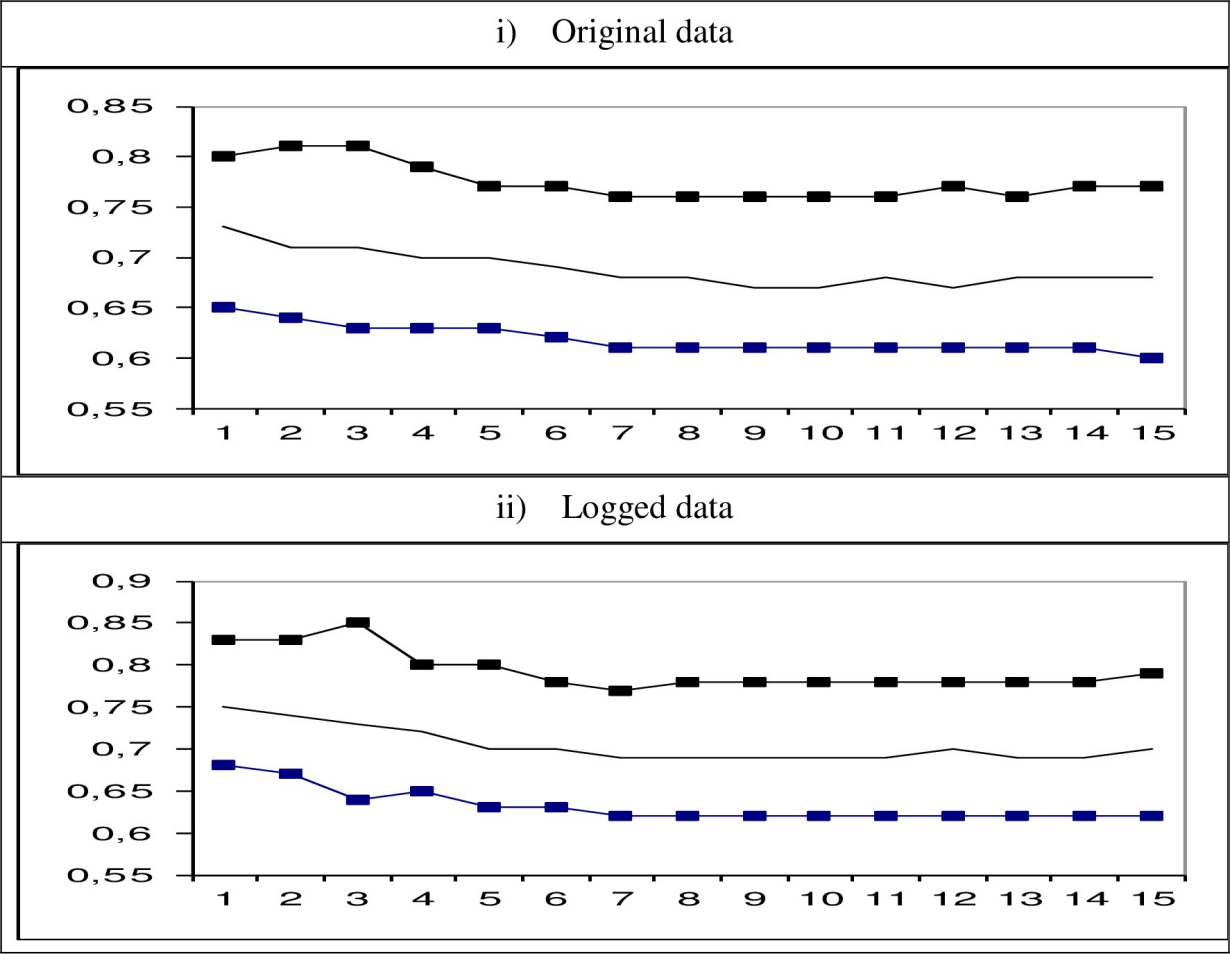
Estimates of d adding successively one observation since February 2020.

We have finally estimated recursively d, first with data ending at February 2020 and then adding successively one observation each time until the end of the sample in April 2021. We focus on the case of autocorrelated (Bloomfield) disturbances, and the results are reported in Fig 3. The upper part refers to the original data while the lower panel to the logged transformed data. In the two cases, we observe a continuous slight decrease in the degree of integration of the series, moving from 0.73 to 0.68 with the original data, and from 0.75 to 0.70 with the logged values. Thus, it seems that the COVID-19 pandemic has not increased the degree of persistence of the series but just the contrary, reducing slightly its dependence. Thus, shocks are transitory, long lived but as time goes by the recovery seems to be faster.

## 5. Conclusions

With such positive numbers, we can talk about how the car industry continues to evolve and invests to prolong the product life cycle with electrical and more intelligent cars. Another factor that helps to develop the industry is all the suppliers and other dependent industries (wheels, batteries, aluminium, etc.), as these companies also are in contact with and evolve with the market. A further additional reason to support this positive situation is that having a car is a symbol in society and a need in developed countries.

In this paper we have adopted a fractional integration approach in order to analyse the sales in the automobile industry in the US. Our results indicate that the fractional differencing parameter belongs to the range [0.5, 1] supporting fractional integration, nonstationarity and mean reverting behaviour, with shocks having long-lived effects though disappearing in the long run. Note that impulse responses can be computed for the cases of white noise and monthly AR(1) errors but not for the exponential spectral model of Bloomfield [37]. In any case, the fact that d is significantly smaller than 1 guarantees the reversion to the mean. The COVID-19 pandemic has had almost no effect and, if any, has reduced slightly the degree of dependence in the data.

The fact that the series seems to be mean reverting has important implications. Thus, for example, in case of a negative shock there is no need for extreme actions since the series will return to its trend sometime in the future. On the other hand, if the shock is positive, actions will be required to maintain this new level/trend in the series.

The results reported in this work can be extended in various directions. Thus, for example, the evidence found in favour of fractional integration may be confirmed by using other parametric or even semiparametric methods. In addition, the presence of breaks or non-linearities in the data can also produce spurious evidence of long memory. In that respect, other statistical methods that allow for break and/or non-linear structures (e.g,. [38, 39]) can be employed in these or in other similar time series data.
